# Effects of a Powder Formulation of *Streptomyces cameroonensis* on Growth and Resistance of Two Cocoa Hybrids from Cameroon against *Phytophthora megakarya* (Causal Agent of Black Pod Disease)

**DOI:** 10.4014/jmb.2110.10006

**Published:** 2021-12-22

**Authors:** Dzelamonyuy Aristide, Tene Tayo Paul Martial, Ngotcho Ngassam Esther Ruth, Lele Brenda Grace, Foka Tatiekam Ebenezer, Magni Pacha Tatiana Flore, Boudjeko Thaddee

**Affiliations:** 1 Laboratory of Phytoprotection and Plant Valorization, Biotechnology Center, University of Yaoundé I P.O. Box 17673 Etetak-Yaoundé, Cameroon; 2 Department of Biochemistry, Faculty of Science, University of Yaoundé 1, P.O. Box 812, Yaoundé, Cameroon

**Keywords:** *Streptomyces cameroonensis*, bioformulation, cocoa, *Phytophthora megakarya*

## Abstract

In the present study we evaluated the efficacy of a bioformulation of *Streptomyces cameroonensis* for control of black pod disease in cocoa and enhancement of seedling growth. The formulation developed using talc powder and cassava starch as carriers showed high shelf-life of 1.07 × 10^6^ CFU/g after six months storage at 4°C. The formulation was tested for inhibition of spore germination in *Phytophthora megakarya* and showed 100% inhibition at 10% (w/v) of formulation. To determine the efficacy of the formulation, we performed an *in planta* assay in the greenhouse on two hybrids of cocoa seedlings, the tolerant SNK413 × (♂) T79/467 and the susceptible UPA 134× (♂) SCA 12. Detached leaf assay showed a significant reduction in the disease severity index of about 67% for the tolerant hybrid and 55% for the susceptible hybrid compared to non-treated plants. A significant enhancement in stem length, leaf surface area and root weight was observed. Analysis of biochemical markers of defense showed a significant increase in total polyphenol, flavonoid, and total protein contents. There was also significant upregulation of PR-proteins such as chitinases, peroxidases and β-1, 3-glucanases following treatment of both tolerant and susceptible hybrids, though with a higher level of synthesis in the tolerant hybrids. A significant increase was also observed in polyphenol oxidase activities in plants treated with the formulation. This work demonstrated the stability and effectiveness of the *S. cameroonensis* powder formulation in suppressing black pod disease in cocoa and subsequently enhancing the growth of seedlings.

## Introduction

Cocoa (*Theobroma cacao* L.) is one of the main cash crops in tropical countries, and in Cameroon its production contributes to the national GDP by 1.2%, generating annual revenue of over €400 million and providing more than 400,000 jobs [1]. However, cocoa cultivation has been confronted by poor yields of the farmed varieties, unavailability of healthy seedings and parasitic attacks such as black pod disease caused by the soil-borne fungal pathogen *Phytophthora megakarya* [2].

In Cameroon, black pod disease stands out as one of the most economically destructive diseases of cocoa [3]. Annual losses are estimated at 40% and could potentially reach up to 90% if no proper control measures are taken [4]. Seedling production is a key step in the production chain of cocoa and in the establishment of new plantations [5]. Attacks of disease at nurseries and farms can lead to unavailability of healthy seedlings.

Countering this problem often requires the use of chemical fungicides but the latter creates many additional problems including the emergence of resistant pathogens, biodegradation of soil, and concerns about human health that constitute a separate dimension to the predicament [6]. However, hybridization has been shown to be a reliable method of ameliorating cocoa production. In Cameroon, research has been initiated towards genetic control by breeding cocoa cultivars less susceptible to the disease and exploiting field resistance factors to find more resistant hybrids with quality seedlings. Several parental cocoa clones, with different sensibility to *P. megakarya* and available in gene banks of the Cameroon Cocoa Development Corporation (SODECAO), for example SNK413 and T79/46, have been bred and analyzed and some of the progenies have demonstrated tolerance to pathogen attack while also showing production of defense markers that complement the tolerance [7]. However, it has been difficult to find a balanced mechanism between disease-resistant hybrids with higher quality seedlings. It is therefore necessary to explore alternative methods of control that are both environmentally safe and effective.

In recent decades, the prospects of phytoprotection through biological control have been regularly explored in the eradication of soil-borne disease [8], and its use is extensive in modern- day agriculture. Intensive research on plant growth-promoting rhizobacteria (PGPR) is being conducted worldwide to develop biofertlilizers and biocontrol agents [9]. Among bacteria communities, Actinobacteria have been reported to comprise several biocontrol agents that suppress plant disease. Actinomycetes produce about 45% of the antibiotics currently in use, among which the genus *Streptomyces* alone produces 73% of the metabolites known to be capable of suppressing plant diseases [10]. *Streptomyces cameroonensis*, an actinomycete isolated from the *Chromolaena odorata* rhizosphere in Yaoundé (Cameroon), has been shown to exhibit extensive antimicrobial effects against a wide range of microorganisms while also possessing PGPR-like traits [11]. This strain demonstrated strong abilities to promote plant growth and protect against *P. megakarya*, the main causal agent of black pod disease during assays performed on cocoa plantlets. The mechanism of disease suppression by *S. cameroonensis* involves production of antibiotics like geldanamycin, production of cell wall-degrading enzymes, hyperparasitism, and production of volatile compounds, competition, and induction of host resistance. This strain is an equally effective root colonizer and can improve plant growth by enhancing iron availability through production of siderophores and production of indole-3-acetic acid and 1-aminocyclopropane-1-carboxylate deaminase activity, nitrogen fixation, and solubilization of phosphates [1]. Having established this microbe as a biocontrol agent, the challenge lies in putting it into forms that are convenient in agricultural systems and better suited for commercial use. Many biocontrol agents have been formulated in various types of powders, granules or liquid. Powder formulations are easy to transport, suitable for easy storage and have longer shelf lives. In addition, the powder formulation can be made into liquid or water-based suspensions for various applications that can involve spraying, root-dipping or seed drenching [9].

In the present study, *S. cameroonensis* isolated from uncropped soil in Yaounde was used in a powder formulation at a nursery in Cameroon to study its shelf-life and efficacy against *P. megakarya* in two hybrids of cocoa as well as its impact on growth and defense-related markers.

## Materials and Methods

### Soil

The soil used was black humus often used by farmers producing cocoa seedlings and was obtained from Nkolbisson, (Yaounde, Centre Region, Cameroon, 3°52′24.4′′N-11°26′7.8′′E). It was dried and sieved to eliminate hard materials and debris, then mixed with river sand obtained from the River Sanaga (Centre Region) at the proportion of 3:1 (w/w).

### Bacterial Strain and Pathogenic Fungi

*S. cameroonensis* was obtained from the microorganism bank of the Laboratory of Phytoprotection and Plant Valorization (LPPV) of the Biotechnology Centre of the University of Yaoundé 1, Cameroon. The strain was cultivated on the International *Streptomyces* Project-2 medium (ISP-2 medium) and incubated at 30°C for 7 days. The bacterial cells were then harvested using glass beads rolled over sporulating colonies. The beads were washed in glycerol (20% v/v), adjusted to 10^9^ CFU/ml and stored at -20°C for further use according to [11].

*Phytophthora megakarya* isolate used in this study was obtained from the microorganism bank of the Laboratory of Phytoprotection and Plant Valorization (LPPV) of the Biotechnology Centre of the University of Yaoundé 1, Cameroon. Zoospore suspensions of *P. megakarya* isolate PM5 were obtained as described previously [12].

### Powder-Based Formulation of *S. cameroonensis*

The powder formulation was developed as described by Anitha and Rabeeth [13] with some modifications. A mixture of 1 kg cassava starch and talc powder were mixed in different proportions (100:0% v/v, 25:75% v/v, 50:50% v/v, 75:25% v/v, 0:100% v/v, respectively) and sterilized twice at 121°C in an autoclave. Upon cooling, the various mixtures were supplemented with 15 g calcium carbonate and 10 g methyl cellulose. The powder mixture was then mixed under sterile conditions with 400 ml of sporal solution obtained previously containing 10^9^ CFU/ml filtered broth of *S. cameroonensis*. The various mixtures were shade dried, ground to powder form and passed through a 0.8 mm sieve to obtain homogenous powder. The resulting powder was then packaged in pre-sterilized polypropylene bags and sealed. The different formulations were then tested for spore viability after 30 days using the spread plate technique. The formulation with the highest spore viability was selected for further testing.

### Study of the Shelf Life of Powder-Based Formulation of *S. cameroonensis*

The formulation with the most viable spore count was stored at 4°C and 25°C, respectively. The shelf lives of the formulation at the two temperatures were determined using the standard dilution plate count method. Samples were taken periodically at 1 month intervals over a period of six months. Three plates were used for each treatment. Humidity, form and texture were evaluated to determine the best conditions of storage as described previously [13].

### Efficacy of *S. cameroonensis* Powder-Based Formulation

The efficacy of the formulation was evaluated in vitro against *P. megakarya*. This was done using the agar well diffusion method. One gram of the powder formulation was dissolved in 1 ml distilled water. PDA media were prepared and supplemented with the water-based suspension of the formulation at various concentrations (50%w/v, 25% w/v, 10 % w/v, 1% w/v and 0.1% w/v). *P. megakarya* was cultured in the middle of the solid PDA media and the percentage radial growth inhibition was calculated after 7 days using the formula:

$$\text{Inhibition (\%)}=\frac{\text{Radial growth of control-Radial growth of treatment}}{\text{Radial growth of control}}\times 100.$$

### Seed Treatment Using *S. cameroonensis* Powder-Based Formulation

Two hybrids of cocoa, (H1 (♀) SNK413 × (♂) T79/467) and H2 ((♀) UPA 134× (♂) SCA 12) produced by manual pollination, were obtained from the SODECAO (“Société de Développement du Cacao”) experimental farm in Mengang, South Region, Cameroon. The cocoa pods were dehusked and the seeds washed with sand and distilled water to remove the mucilage. The washed seeds were surface sterilized with 70% ethanol for 5 min and then later with 0.2% sodium hypochlorite solution for another 5 min. The sterilized seeds were then rinsed with sterilized distilled water to prepare a 0.1 g/ml water suspension of the formulation. The cocoa seeds were divided into three groups. The first group was soaked in the formulation suspension, the second group in distilled water and the third group in a suspension of the chemical fungicide MANCOXYL PLUS 720WP. The soaked seeds were incubated at 150 rpm at 28°C for 24 h prior to planting.

### Production of Cocoa Plant Seedlings under Greenhouse Conditions and Evaluation of Agro-Morphological Parameters

The treated seedlings were then planted in pots containing soil and river sand mixed at the ratio of 3:1. Each treatment was in duplicates of 60 seedlings. The pots were kept in a greenhouse and watered with distilled water every two days for a period of 12 weeks. Stem length, leaf number and leaf surface area were measured every 4 weeks. At the end of the 12^th^ week, the dry and fresh weight of roots and shoots were also measured. The experiment was a completely randomized design with 3 treatments per hybrid, formulation-treated seedlings (H1T and H2T), non-treated seedlings (H1NT and H2NT) and chemically-treated seedlings (H1C and H2C).

### Assessment of Induced Resistance

The formulation was assessed for its efficacy in controlling the incidence of *P. megakarya* using detached leaf inoculation test [5] with some modifications. Briefly, young leaves were detached from cocoa seedlings in the greenhouse after 12 weeks of growth. The leaves were washed with distilled water and surface sterilized with ethanol (70% v/v) for 30 s. For each treatment, leaves were divided into two, one group for the inoculation with 10 μl of 10^6^ zoospore/ml suspension of *P. megakarya* on the underside leaf surface while the other group for the control was inoculated with an equivalent amount of sterilized distilled water. The inoculated leaves were incubated in a humid dark chamber at 25 ± 1°C. Each treatment consisted of three replicates of 6 leaves each. Disease expression was rated six days after and by using Nyassé’s rating scale [14]. This experiment was repeated twice and the severity of disease was determined for each treatment by calculating the ratio of the sum of individual scores to the total number of leaves used. The disease severity index used to express the resistance level [15] was as follows: Highly Resistant (HR: 0 < index ≤ 1); Resistant (R: 1 < index ≤ 2); Moderately Resistant (MR: 2 < index ≤ 2.5); Susceptible (S: 2.5 < index ≤ 3.5); and Highly Susceptible (HS: 3.5 < index ≤ 5).

### Biochemical Analysis

Biochemical analyses were carried out by assessing the level of infections on whole detached leaves after seven days of inoculation with *P. megakarya* sporal solution. The samples involved were cut at about 1 cm from beyond the necrosis point where no symptoms were noticed. For biochemical analyses, each treatment was repeated twice.

### Determination of the Content of Total Phenolic Compounds and Flavonoids

The extraction of phenolic compounds was done following the modified protocol [4]. One gram of tissue extract (leaf) was ground in 5 ml methanol 80% (v/v). The sample was incubated at 4°C, and centrifuged at 10,000 ×*g* for 5 min at room temperature using the Beckman-Coulter Microfuge 20R centrifuge. The supernatant was collected and the precipitate re-suspended in 3 ml methanol, and incubated at room temperature for 15 min followed by further centrifugation. The second supernatant was collected and mixed with the first to constitute the extract.

Concentration of phenolic compounds was determined spectrophotometrically at 725 nm according to the method of [16], using the Folin-Ciocalteu reagent. Total content of phenolic compounds was expressed in milligrams of gallic acid equivalents per gram of fresh weight. Flavonoid content was determined in phenolic extract according to the method described by Kramling and Singleton [17] with some modifications. Briefly, 400 μl of phenolic extract, 200 μl of HCl (50%) and 200 μl of formaldehyde (8 mg/l) were incubated 15 min at 4°C and centrifuged at 3,000 ×*g* for 5 min at 4°C. The supernatant was collected and used for non-flavonoid quantification spectrophotometrically at 725 nm [16].

### Determination of Total Protein Content

For the determination of total protein content, extraction was performed as described by [13] with some modifications. One gram of plant samples (inoculated and healthy leaves) were crushed separately in a pestle and homogenized in 5 ml of the Tris-Maleate buffer (Tris-cHcl 1 HCl 10 mM, Triton X-100 1%, pH 7.5) at 4°C. The homogenate was centrifuged at 10,000 ×*g* for 25 min at 4°C using the Beckman-Coulter Microfuge 20R centrifuge. The supernatant was collected and the precipitate re-suspended in 3 ml buffer followed by further centrifugation. The second supernatant was collected, mixed with the first in 1.5 ml Eppendorf tubes and stored at -20°C. Quantification of the concentration of total proteins was done using the standard Bradford assay. The absorbance was measured at 595 nm using a UV-Vis 1605 Shimadzu spectrophotometer. For each extract, three repetitions were carried out. Bovine Serum Albumin was used as the standard. The concentration of the protein present was expressed in mg/g of fresh matter.

### Evaluation of the Enzymes Activities

Peroxidase activity was assayed spectrophotometrically at 470 nm in the total protein extract [4]. The enzymatic activity was expressed in enzymatic units per gram of fresh weight (Δ470/min (UE)/g of Fresh Weight). Polyphenoloxidase activity was assayed spectrophotometrically [18], using catechine as a substrate. Enzyme activity was expressed as “A330 nm/min/g fresh weight”. Chitinase activity was determined by colorimetric assay using bipolymeric substrate colloidal Chitin-RBV [19]. Chitinase activity is expressed in unit/g fresh matter/h. One unit of chitinase activity corresponds to an increased absorbance of 0.1. The β-1,3-glucanase activity was assayed using laminarin as a substrate. The quantity of glucose released was determined spectrophotometrically as changes in absorbance were measured at 540 nm. The amount of reducing sugars released was calculated from a standard curve prepared with glucose and the glucanase activity was expressed in units (μg glucose/min/mg protein).

### Statistical Analysis

All experiments were conducted in triplicates and all data were expressed as means ± SD and subjected to one-way ANOVA. Tukey’s test and probability values of *p* ≤ 0.05 were considered significant using the Statistix software version 9.0.

## Results

### Dry Formulations of *S. cameroonensis*

**Fig. 1:**
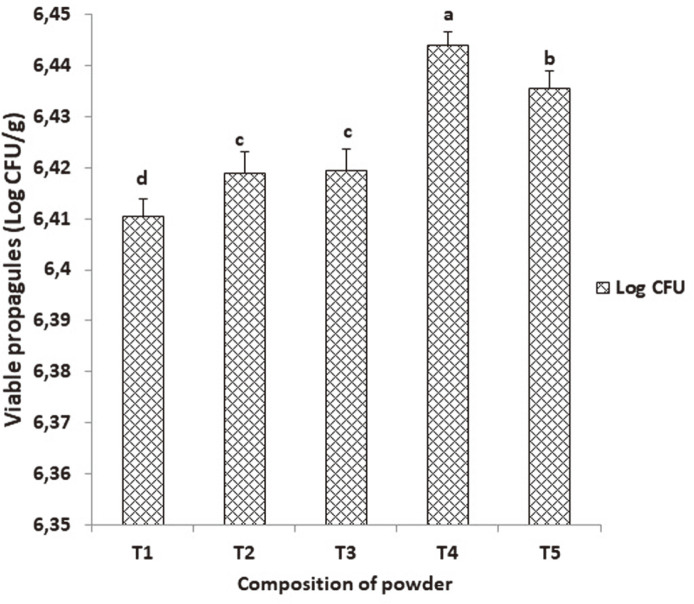
*S. cameroonensis* spore viability in various proportions of cassava starch/talc powder after 1 month. *Values with the same letter within a column are not significant at *p* ≤ 0.05. Note: T1: 0%talc/100% w/w Cassava starch; T2: 25%talc/175% w/w Cassava starch; T3: 50%talc/50% w/w Cassava starch; T4: 75%talc/25% w/w Cassava starch; T1: 100%talc/0% w/w Cassava starch.

Dry powder formulations of cassava starch and talc powder with the percentage proportions of 25/75% (w/w) yielded the maximum population of bacterial colonies (2.78 × 10^6^ CFU/g) than the rest of the proportions (Fig. 1). The concentration of the cassava starch influenced the viability of the spores at a concentration of 25% while the talc powder concentration in the formulation supported the maximum spore viability at 75% more than at 100%. This particular formulation produced the finest powder.

### Shelf Life Stability of *S. cameroonensis* in Starch/Talc-Based Powder Formulation

**Fig. 2:**
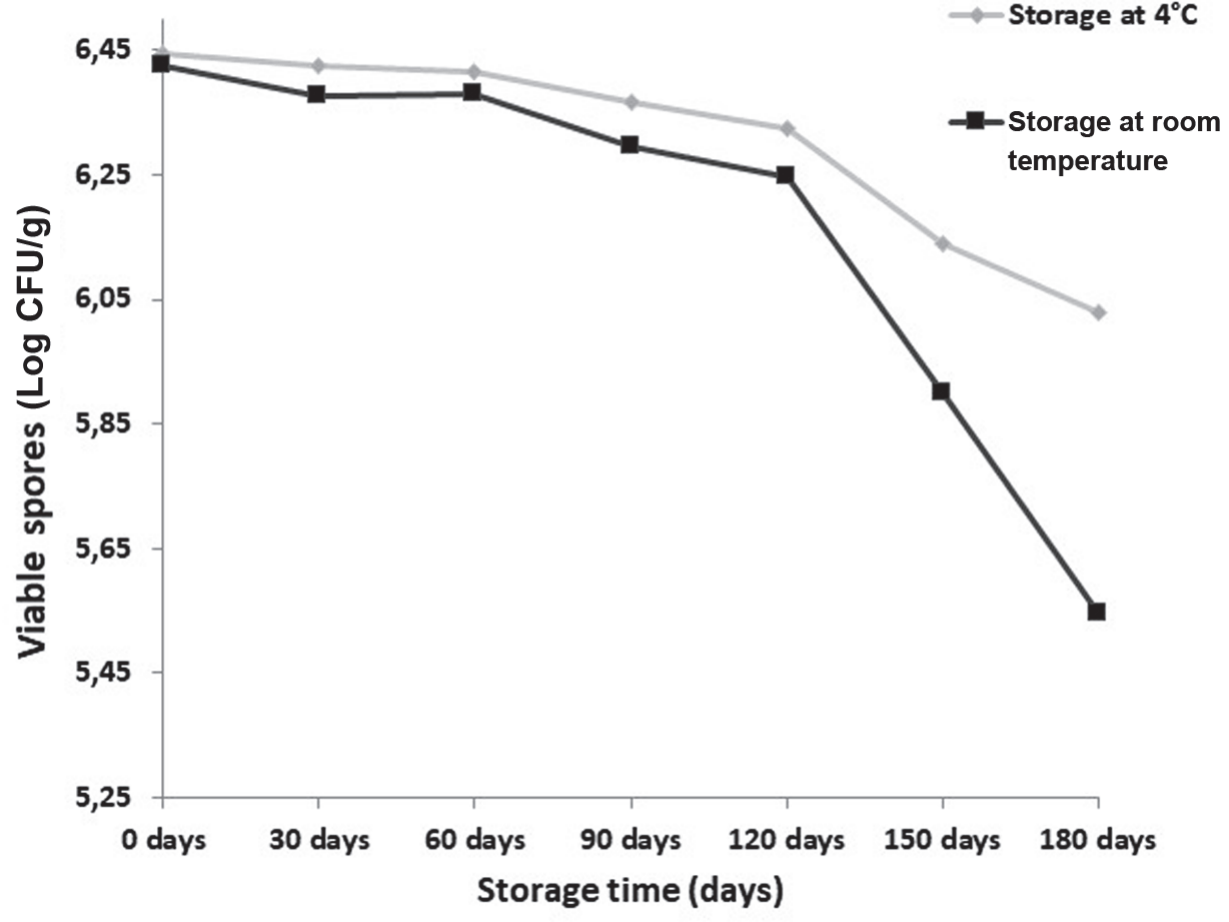
Shelf-life of the cassava starch/talc powder formulation of *S. cameroonensis* at different temperatures.

The shelf life of the formation remained stable after 6 months. The formulation stored at 4°C showed longer shelf life than the formulation stored at 25°C. After 180 days, cell count in powder formulation stored at 4°C was 1.07 × 10^6^ CFU/g compared to 3.53 × 10^5^ CFU/g for the formulation stored at 25°C (Fig. 2).

### Biocontrol Efficacy of *S. cameroonensis*-Based Powder Formulation

**Fig. 3:**
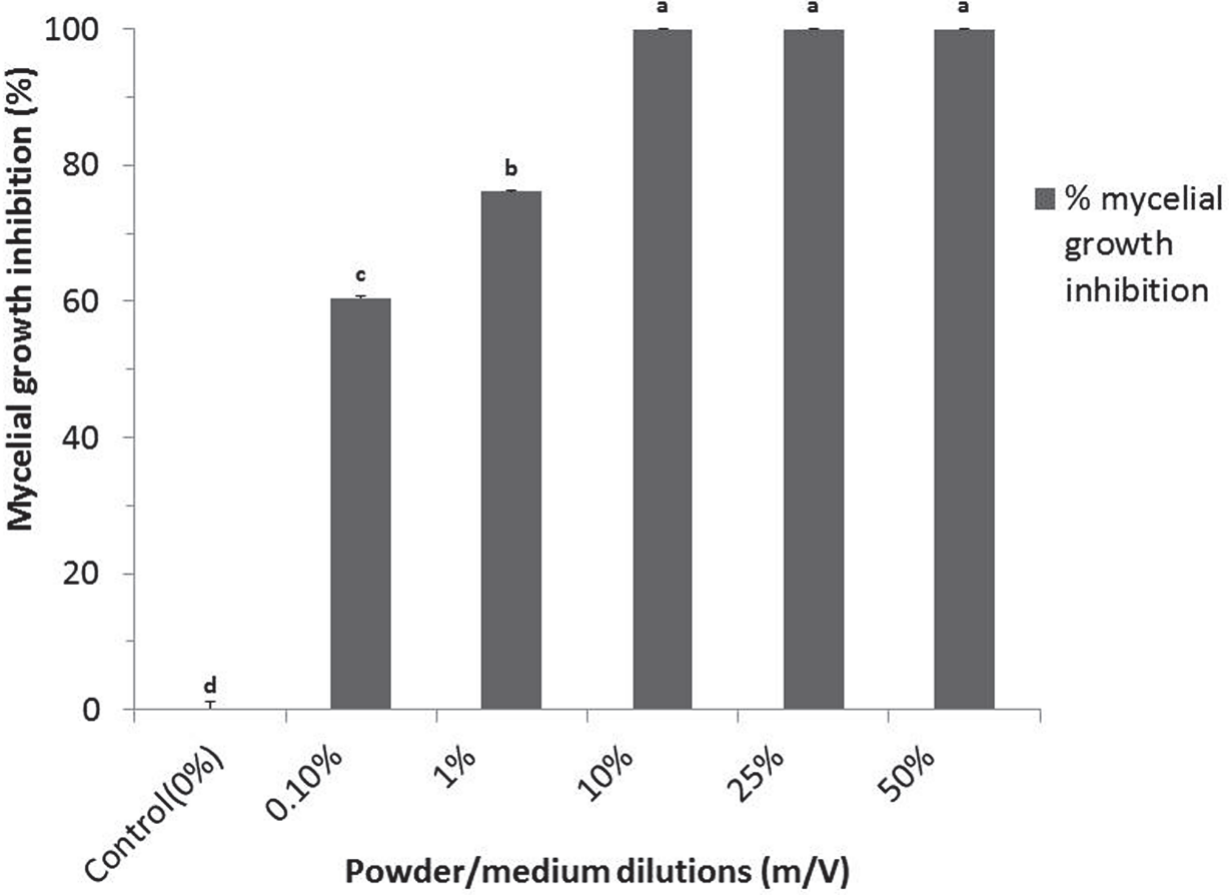
Mycelial growth inhibition of fungal pathogen *Phytophthora megakarya* at various concentrations of the powder formulation. *Values with the same letter within a column are not significant at *p* ≤ 0.05. Note: **0% (Control):** 0% formulation; **0.1%:** 0.1%w/v formulation concentration; **1%:** 1% w/v formulation concentration; **10%:** 10% w/v formulation concentration; **50%:** 50% w/v formulation concentration; **100%:** 100% w/v formulation concentration.

The formulation powder exhibited a high biocontrol efficacy (Fig. 3). At very low concentrations of 10% (w/v), the formulation exhibited a 100% mycelial inhibition against *P. megakarya*. The percentage mycelial growth inhibition at very low concentrations of 1% (w/v) and 0.1% (w/v) were at 76.2% and 60.5 %, respectively.

### Growth Promotion under Greenhouse Conditions

Formulation-treated plants showed significant increase (*p* ≤ 0.05) in plant height, number of leaves, leaf surface area, fresh and dry weight of shoots and roots over the non-treated and chemically treated plants after 12 weeks of growth (Table 1). The tolerant hybrid (H1: (♀) SNK413 × (♂) T79/467) showed a greater degree of growth than the susceptible hybrid (H2: (♀) UPA 134× (♂) SCA 12) in all cases of treatment. Plant height in formulation-treated plants for H1 was significantly higher (37.30 ± 0.81 cm) compared to non-treated plants (30.4 ± 0.74 cm). The same trend was observed for H2 in the order of 26.43 ± 0.74 cm and 23.76 ± 0.24 respectively for formulation-treated (T) and non-treated (NT). The same trend was observed for leaf surface area (Table 1). The dry and fresh weight of roots and shoot for both hybrids showed a significant increase in formulation-treated than the non-treated and chemical treatments. However, H1 showed higher levels of dry and fresh weight than H2.

### Assessment of Disease Severity on Inoculated Leaves

**Fig. 4:**
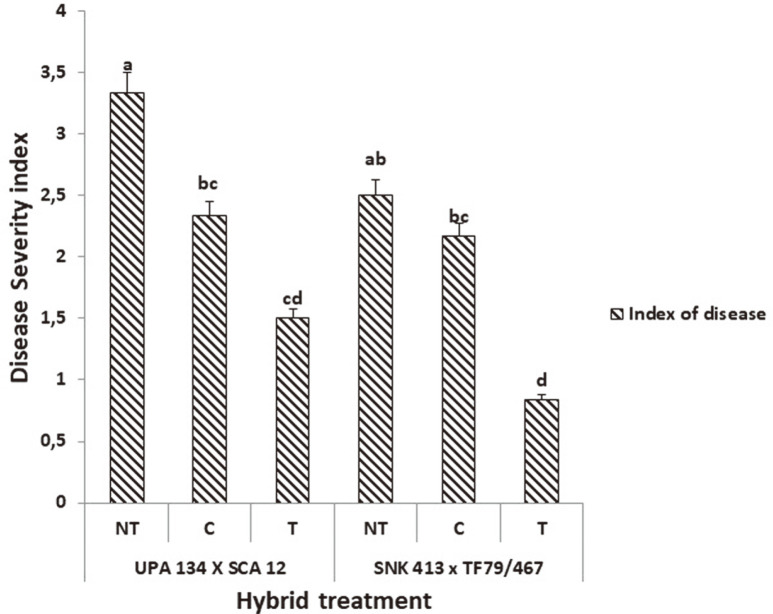
Disease severity index of plants under different treatments six days after inoculation. *Values with the same letter within a column are not significant at *p* ≤ 0.05. Note: **NT:** Non-treated (negative control); **T:** Treated (with bioformulation); **C:** Chemically treated (With MANCOXYL PUS 720WP).

Necrotic lesions were noticed on all leaves inoculated with the *P. megakarya* inoculum six days after inoculation while no lesions were found on leaves inoculated with sterilized distilled water. A significant lowest disease severity index was noticed on plants treated with the *S. cameroonensis*-based formulation compared to the non-treated for both hybrids with about 67% for H1 and 55% for H2 (Fig. 4). This led us to classify the H1 plants treated with the formulation as highly resistant with a necrosis index of 0.83, while the H2 plants treated with the formulation were then classified as resistant with a necrosis index of 1.5. The chemically treated plants were classified as moderately resistant with a necrosis index of 2.1 and 2.3 for H1 and H2, respectively. The non-treated H1 plants also showed moderate resistance (necrosis index of 2.5) while the non-treated H2 plants were classified as susceptible with a necrosis index of 3.3.

### Biochemical Analysis

**Fig. 5:**
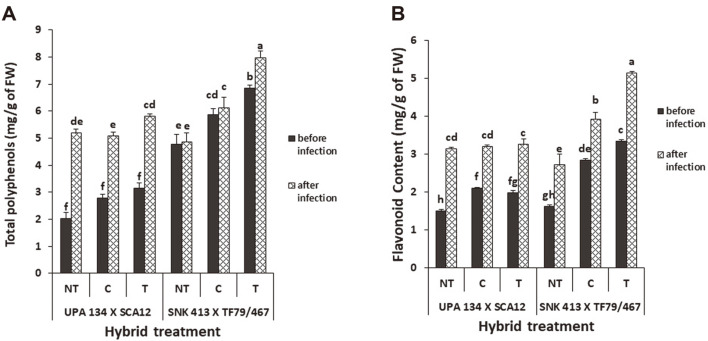
Total polyphenol content (a) and flavonoid content (b) in *S. cameroonensis* based powder formulation treated and non-treated plants before and after inoculation. *Values with the same letter within a column are not significant at *p* ≤ 0.05. Note: NT: Non-treated (negative control); T: Treated (with bioformulation); C: Chemically treated (With MANCOXYL PUS 720WP).

**Total phenol and flavonoid content.** The *S. cameroonensis*-based formulation-treated plants showed a significant increase in total phenol content as compared to the chemical control and the non-treated plants. H1 also showed a greater difference in phenol content than H2 by 40% after inoculation for the formulation-treated plants (Fig. 5A). Formulation-treated plants showed a percentage increase of 16% for H1 and 85% for H2 after inoculation while the chemically treated showed an increase of 5% and 83% respectively for both hybrids, H1 and H2. Flavonoid content in inoculated leaves was significantly higher than in the non-inoculated leaves for both hybrids. Formulation-treated H1 plants showed a significantly higher flavonoid content than chemically treated (31%) and non-treated plants (88%) before and after inoculation, while that for H2 was not significant. (Fig. 5B)

**Fig. 6:**
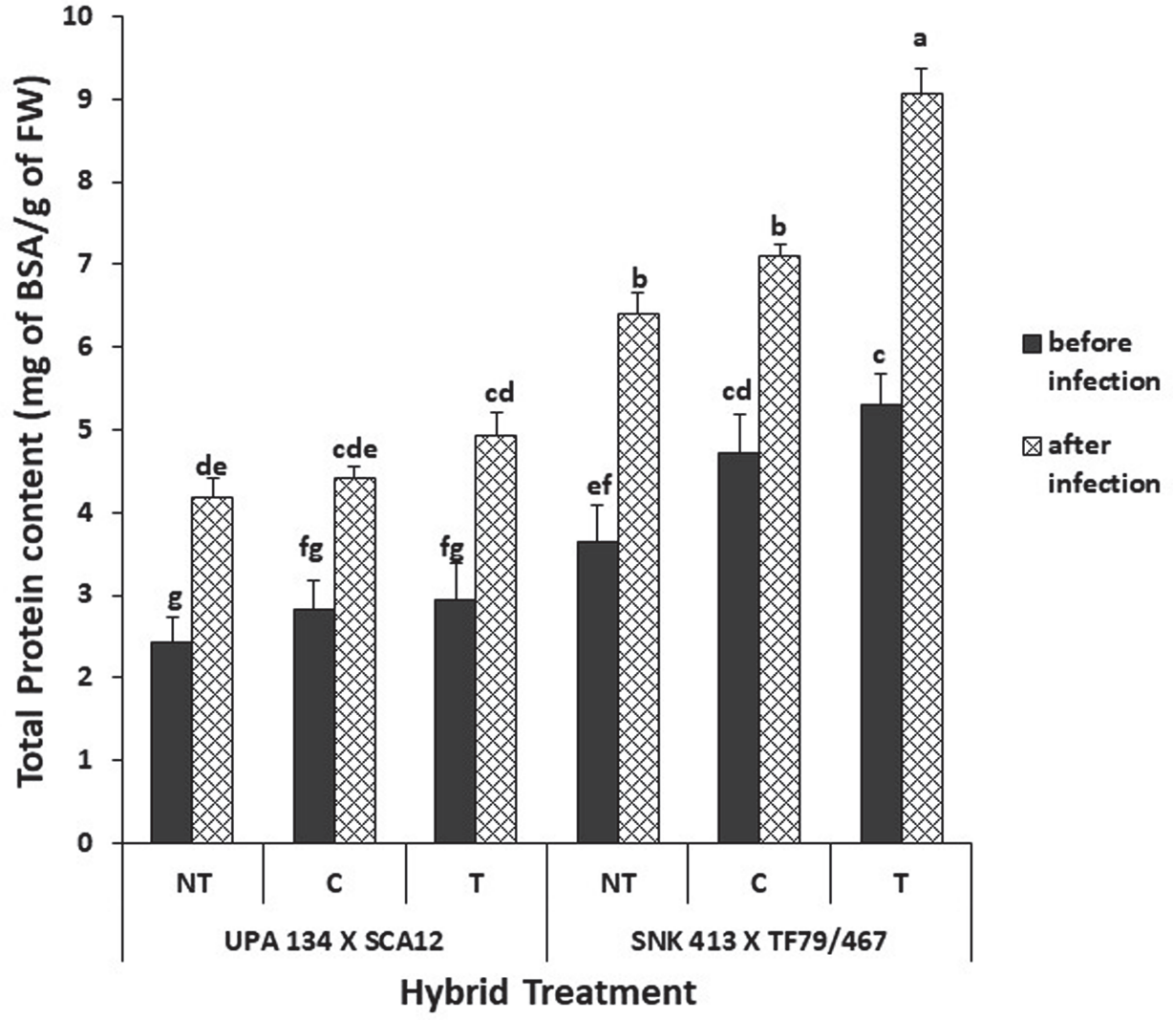
Total protein content in *S. cameroonensis* based powder formulation treated and non-treated plants before and after inoculation. *Values with the same letter within a column are not significant at *p* ≤ 0.05. Note: NT: Nontreated (negative control); T: Treated (with bioformulation); C: Chemically treated (With MANCOXYL PUS 720WP).

**Total protein content.** Formulation-treated plants showed a significantly higher protein content than plants chemically treated and non-treated for both hybrids H1 and H2 (Fig. 6). H1 however showed a higher protein content after inoculation equivalent to 5.30 mg equivalent of BSA/g of fresh weight (FW) and 2.94 mg equivalent of BSA/g of fresh weight for H2, which rose significantly by 71% and 67% respectively after inoculation.

### Enzymatic Activities

**Fig. 7:**
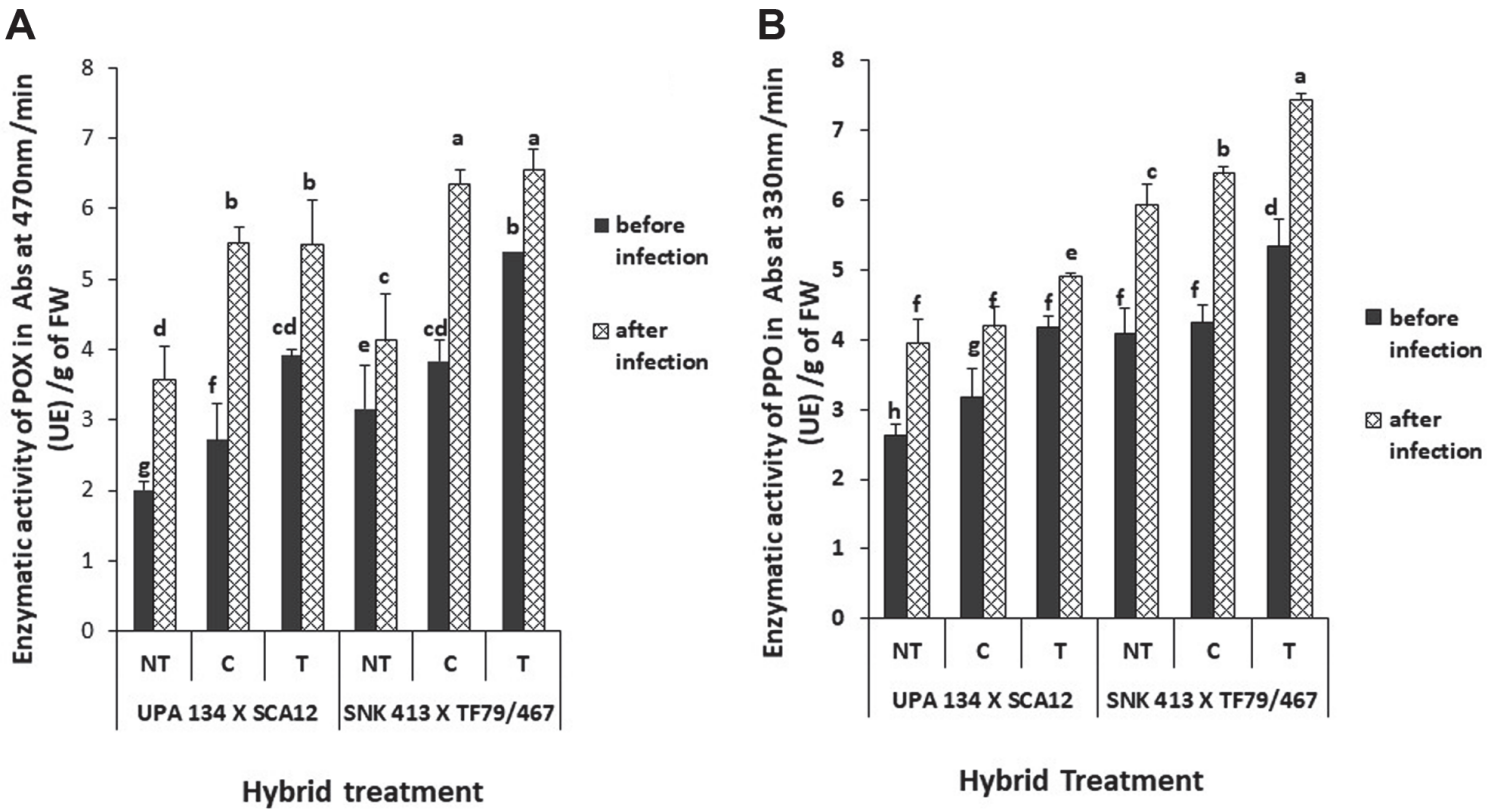
Enzymatic activity of peroxidase (**A**) and polyphenoloxidase (**B**) in *S. cameroonensis* based powder formulation treated and non-treated plants before and after inoculation. *Values with the same letter within a column are not significant at *p* ≤ 0.05. Note: NT: Non-treated (negative control); T: Treated (with bioformulation); C: Chemically treated (With MANCOXYL PUS 720WP).

**Fig. 8:**
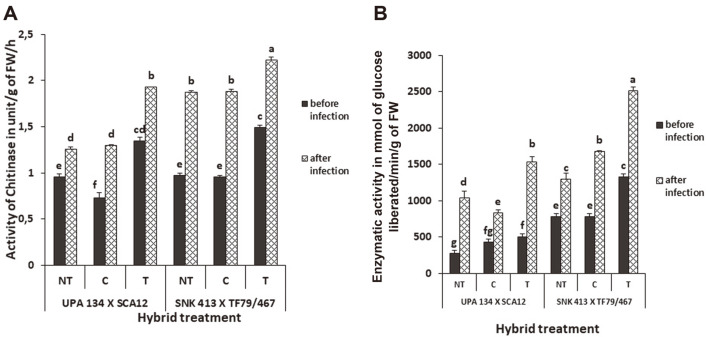
Enzymatic effect of Chitinase (**A**) and β-1,3-glucanase (**B**) in *S. cameroonensis* based powder formulation treated and non-treated plants before and after inoculation. *Values with the same letter within a column are not significant at *p* ≤ 0.05. Note: NT: Non-treated (negative control); T: Treated (with bioformulation); C: Chemically treated (With MANCOXYL PUS 720WP).

Formulation-treated plants had a higher peroxidase activity than the chemically treated and the non-treated plants. In H1, the formulation-treated plants showed a significantly higher peroxidase activity (5.34 UE/g FW) than the chemically treated (4.25 UE/g FW) and the non-treated (4.08 UE/g FW). The activity of peroxidase effectively rose by 39%, 50% and 45%, respectively, after inoculation (Fig. 7A). H2 showed a similar pattern but with generally less UE/g of fresh weight before and after inoculation. Polyphenoloxidase activities were higher in the formulation-treated plants than the controls before and after inoculation. In H1, the formulation-treated plants showed a significantly higher polyphenoloxidase activity (5.34 UE/g FW) which rose by 39% after inoculation. H2 showed a lower activity (4.17 UE/g FW), which rose by 18% after inoculation (Fig. 7B). There was a significant increase in the activity of chitinase in formulation-treated plants compared to the chemically treated plants and non-treated plants. In H1, the activity of chitinase for formulation-treated plants stood at 1.49 UE/g of fresh weight and rose by 49% after inoculation. H2 followed a similar pattern with 1.35 UE/g of fresh weight that rose to 43% after inoculation (Fig. 8A). As shown in Fig. 8B, formulation-treated plants (T) had a significant increase in β-1, 3-glucanase production as compared to the non-treated (NT) and chemically treated (C). There was a general significant increase in β-1, 3-glucanase after inoculation for both hybrids and all treatments. The activity of β-1, 3-glucanase increased by 89% after inoculation for formulation-treated H1 plants while that of H2 increased more than 200% (Fig. 8B).

## Discussion

In this present study, *S. cameroonensis*, an actinomycete isolated from uncropped soil in Yaoundé, has been shown to survive well in a mixture of talc/cassava starch powder-based formulations (75%/25% w/w) for more than 6 months at 4°C as well as at room temperature. *Streptomyces* species and other plant growth-promotion rhizobacteria (PGPR) have been shown to survive in dry formulations including talc, corn starch, xanthan gum, and peat [9, 13, 20]. The talc and cassava starch suitable in enhancement of Actinobacteria cell survival is probably related to the nature of their lipopolysaccharides and coating properties. Talc/cassava starch formulation of *S. cameroonensis* strongly suppressed the growth of *P. megakarya* in vitro even at very low concentrations in PDA medium. Boudjeko *et al*. [11] showed that *S. cameroonensis* possessed antifungal properties by producing bioactive molecules like geldanamycin which has the ability to inhibit growth of oomycetes. *Streptomyces* species are also known to produce major fungal degrading cell wall enzymes such as chitinase and β-1,3-glucanase which tend to inhibit mycelial growth of fungal pathogens [9, 21]. These results are in agreement with our findings.

The efficacy of this formulation was tested on two hybrids of cocoa commonly used in Cameroon, H1: SNK413xTF79/467 (tolerant) and H2:UPA134 X SCA 12(susceptible) [22]. The formulation enhanced significantly the growth of cocoa seedlings of the two hybrids under greenhouse conditions and greatly reduced the severity of *P. megakarya* infection on leaves of cocoa inoculated with *P. megakarya* in vitro. Boudjeko *et al*. [11] previously demonstrated that *S. cameroonensis* possess growth-promoting properties such as production of siderophores and indole acetic acid, solubilization of phosphates, and degradation of 1-aminocyclopropane-1-carboxylate, an intermediary product in the biosynthetic pathway of the plant hormone ethylene. The production of these substances has been reported in several cases where treatment with *Streptomyces* species has enhanced growth of *Vigna unguiculata* and *Arabidopsis* in vitro [23, 24].

Both hybrids exhibited a low disease severity index after treatment with the *S. cameroonensis*-based powder formulation. Boudjeko *et al*. [11] previously reported that the necrosis index for cocoa foliar discs inoculated with sporal solution of *P. megakarya* for plants grown in substrate inoculated with *S. cameroonensis* reduced significantly compared to the control by stimulating systemic resistance in cocoa plants. Given that the powder formulation was applied as seed treatment by enrobing cocoa grains, when the treated seeds are sown, the bacteria will establish well into the seed surface and colonize the roots after germination. Previous studies have shown that a talc-based powder formulation of *Pseudomonas fluorescens* when applied as seed treatment controlled foliar infection of rice blast and could be detected on the root cortex of leave sheaths [20]. Talc-based formulations of *Streptomyces griseus* suppressed *Fusarium* wilt of tomato in greenhouse [13]. Similarly, *Streptomyces corchorusii* formulated on talc strongly enhanced the growth of rice plants under pathogen-challenged conditions and suppressed root disease caused by *Macrophomina phaseolina* under greenhouse conditions [9, 25]. This antifungal activity could as well be attributed to the production of antimicrobial compounds like geldanamycin or the production of many fungal cell wall- degrading enzymes.

The increase in resistance was translated by the increased synthesis of biochemical markers of defense. The level of disease severity was lower in hybrid H1, which was the more tolerant and displayed higher levels of phenol content, flavonoid content, protein content and increased activities of polyphenoloxidase, peroxidases, chitinase and β-1,3-glucanases. Previous studies have shown that the tolerant and more productive species displayed high phenol content while less tolerant ones displayed less phenol content [26]. The role of phenolic compounds and flavonoids in plant defense is well documented [27] as these metabolites tend to accumulate in different levels in infected tissues in response to pathogen attack. The use of *S. cameroonensis* powder formulation led to a significant increase in the synthesis of these metabolites signifying a corresponding increase in growth and resistance against pathogen attack in cocoa seedlings. The activities of defense-related enzymes like PPO, POX, chitinase and β-1,3-glucanase increased higher in *S. cameroonensis* powder formulation-treated plants compared to the controls. Studies have shown that POX is important in scavenging for H_2_O_2_ in cells which is an important element in disease resistance to pathogens. The enhanced activities of these enzymes in plant tissues are positively associated to induce systemic resistance and plant disease suppression [5, 28]. Chitinases and glucanases have been demonstrated by many authors to be fungal pathogen cell wall-degrading enzymes [5]. Previous studies have also reported an increase in activities of these defense-related enzymes as a result of induction by microbial antagonist in hosts [28]. Studies have shown that root inoculation with *Streptomyces* GB 4–2 provided Norway spruce with systemic resistance to the needle pathogenic fungus B. cinerea. Treatment of cucumber leaves with a culture filtrate from *Streptomyces bikiniensis* HD-087 showed an increase in the activities of peroxidase, phenylalanine ammonia-lyase, and beta-1,3-glucanase as well as increased levels of chlorophyll and soluble sugars [30]. These results corroborate our findings that treating seedlings of cocoa with a powder formulation of *S. cameroonensis* induces systemic resistance in the seedlings by causing an increased synthesis of defense markers. Hence, the accumulation of these defense-related enzymes in this study appears as a credible mechanism for the efficacy of this *S. cameroonensis* powder formulation.

In conclusion, the present study clearly demonstrates that *S. cameroonensis* fused in a mixture of talc and cassava starch in the bioformulation performs well in the control of *Phytopthora megakarya*, the causal agent of black pod disease in cocoa. This formulation significantly increased the growth and resistance of two hybrids of cocoa commonly cultivated in Cameroon and significantly so for the more susceptible one by demonstrating an increase synthesis of biochemical markers of resistance. Hence, this formulation could be used as an effective biofungicide for the biocontrol of black pod disease of cocoa. Moreover, based on this present study and other reports, we recommend investigating the genetic basis of the interactions between the formulation and the cocoa seedlings, the concentration of the biocontrol agent in the plant and soil after treatment as well as the follow-up of the treatment to evaluate the effect of the formulation on the final product of cocoa in the farm. This will lay the basis for identifying the upregulated genes corresponding to increased resistance as well as metabolic and transcriptomic profiling and will help us better understand the mode of action of the formulation by studying the plant-bacteria interaction.

## Figures and Tables

**Table 1:** Effect of *S. cameroonensis*-based powder formulation on different growth parameters of cocoa seedlings in nursery after 12 weeks of growth.

Hybrid	Treatment	Leave number/plant	Stem length (Plant/cm)	Leaf Surface area (Plant/cm^2^)	Shoot Fresh weight (g/plant)	Shoot dry weight (g/plant)	Root Fresh weight (g/plant)	Root dry weight (g/plant)
SNK 413 x TF79/467(H1)	NT	12±0.67^ab^	30.4±0.74^b^	56.68±2.19^b^	13.92±0.46^c^	7.14±0.54^ab^	4.69±0.17^bc^	1.80±0.18^cd^
	C	13±0.92^a^	36.00±1.02^a^	58.01±1.59^b^	14.74±0.80^b^	7.56±0.85^ab^	5.19±0.18^b^	2.30±0.11^b^
	T	13±0.74^a^	37.30±0.81^a^	83.78±3.71^a^	16.04±0.54^a^	8.51±0.31^a^	6.52±0.27^a^	3.08±0.14^a^
UPA 134 x SCA 12 (H2)	NT	10±0.47^c^	23.76±0.24^d^	38.23±1.54^d^	10.44±0.38^d^	3.58±0.31^d^	2.83±0.22^e^	1.16±0.14^e^
	C	11±0.83^bc^	24.67±0.94^cd^	43.41±1.78^c^	9.93±0.46^d^	4.33±0.38^cd^	3.34±0.13^d^	1.40±0.10^d^
	T	10±0.83^c^	26.43±0.74^c^	47.50±1.48^c^	10.73±0.57^d^	3.81±0.40^d^	4.27±0.25^c^	1.44±0.09^d^

*Values with the same letter within a column are not significant at *p* ≤ 0.05. **NT:** Non-treated (negative control); **T:** Treated (with bioformulation); **C:** Chemically treated (With MANCOXYL PUS 720WP)
